# Evaluating the Evidence for Neuroprotective and Axonal Regenerative Activities of Different Inflammatory Cell Types After Optic Nerve Injury

**DOI:** 10.1007/s12035-024-04679-3

**Published:** 2024-12-30

**Authors:** Alexander W. Venanzi, Laura D. McGee, Abigail S. Hackam

**Affiliations:** Bascom Palmer Eye Institute, University of Miami Miller School of Medicine, 1638 NW 10Th Ave, Rm 404, Miami, FL 33136 USA

**Keywords:** Optic nerve, Axonal regeneration, Neuroinflammation, Microglia, Muller glia, Macrophage

## Abstract

The optic nerve contains retinal ganglion cell (RGC) axons and functions to transmit visual stimuli to the brain. Injury to the optic nerve from ischemia, trauma, or disease leads to retrograde axonal degeneration and subsequent RGC dysfunction and death, causing irreversible vision loss. Inflammatory responses to neurological damage and axonal injuries in the central nervous system (CNS) are typically harmful to neurons and prevent recovery. However, recent evidence indicates that certain inflammatory cell types and signaling pathways are protective after optic nerve injury and promote RGC survival and axonal regeneration. The objective of this review is to examine the evidence for diverse effects of inflammatory cell types on the retina and optic nerve after injury. Additionally, we highlight promising avenues for further research.

## Introduction

**Fig. 1:**
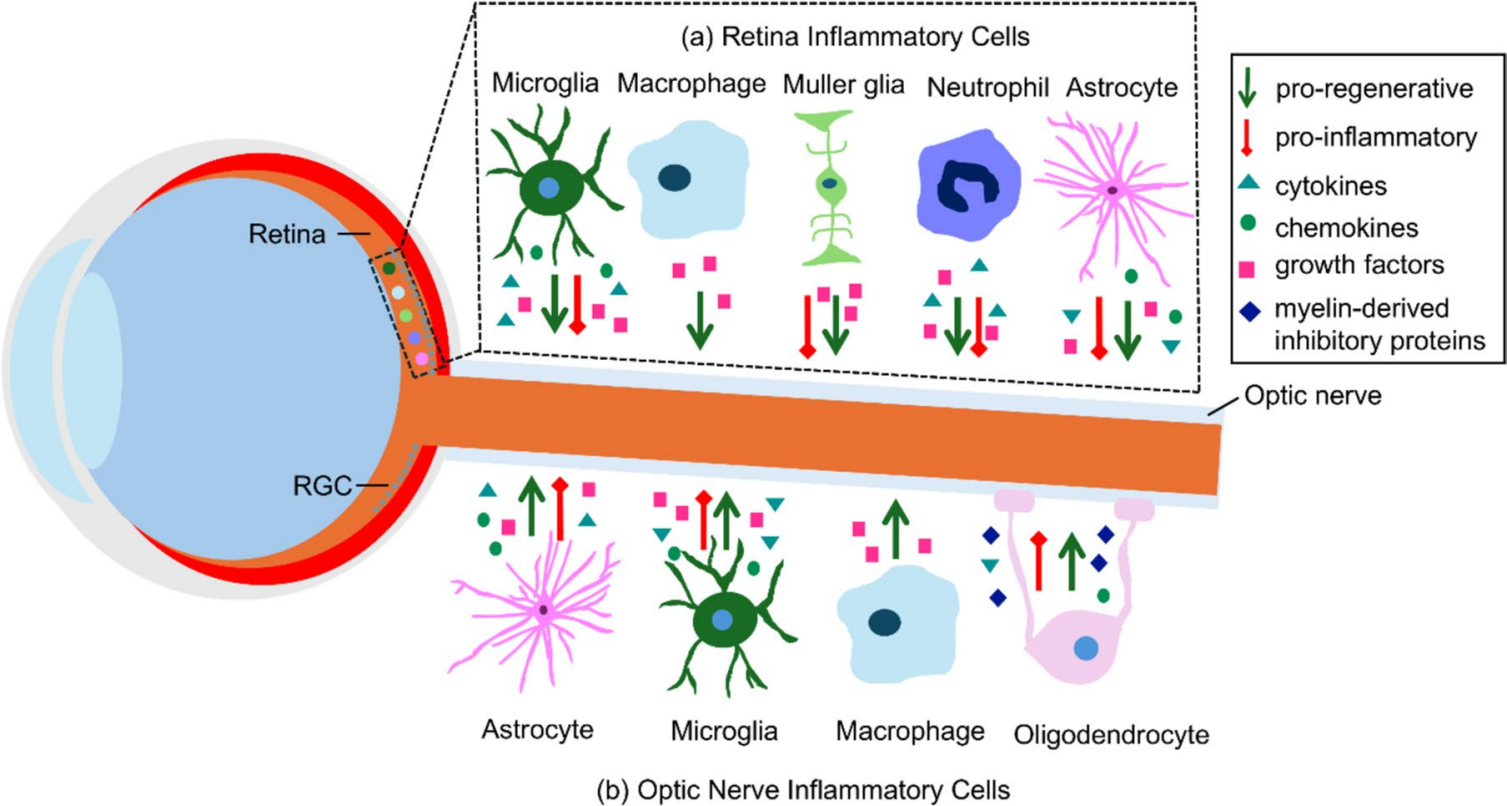
Schematic diagram illustrating different inflammatory cell types within the retina and optic nerve following optic nerve injury. Astrocytes, microglia, macrophages, and neutrophils exist within both the retina (**a**) and optic nerve (**b**), while Muller glia are localized only in the retina (**a**), and oligodendrocytes are limited to the optic nerve (**b**). Secretion of various molecular mediators including cytokines, chemokines, growth factors, and myelin-derived inhibitory proteins modulate pro-regenerative or pro-inflammatory effects. Refer to Table 1 for examples of secreted neuroprotective and regenerative factors by each cell type. Changes in cellular morphologies upon activation are not indicated. See text for further details. Pro-regenerative signaling is indicated by green arrows and pro-inflammatory/neurotoxic signaling is indicated by red lines. RGC: retinal ganglion cell

Neuroinflammation is the general term that describes molecular and cellular inflammatory pathways that are stimulated after neuronal stress, injury, and during disease. The RGCs in the retina are an ideal neuronal type to examine the role of neuroinflammatory responses in degeneration or regeneration because of their well-characterized functions, accessibility, defined anatomical location, and established injury models. RGC cell bodies in the retina, and their axons within the optic nerve, are localized adjacent to multiple inflammatory cell types. These cells include astrocytes, microglia, neutrophils, and macrophages within the retina and optic nerve, Muller glia in the retina, and oligodendrocytes in the optic nerve (Fig. 1).

Numerous RGC injury models in rodents, zebrafish, and other species have been described. The most often used include ischemia–reperfusion, inherited pigmentary and induced glaucoma, retinal excitotoxicity, and optic nerve trauma. These injuries lead to RGC degeneration and secondary axonal atrophy in the optic nerve. A well-characterized model to study inflammatory responses to optic nerve injury and the effects of inflammation on axonal regeneration in the optic nerve is the optic nerve crush model [1]. Optic nerve crush consists of a brief gentle pinching of the optic nerve behind the globe, which leads to neuronal and axonal degeneration [2]. RGC death after optic nerve crush is stimulated by reduced axonal transport, accumulation of myelin and other cellular debris, and loss of cellular homeostasis. Various cell stress responses have been described after optic nerve crush, including mitochondrial dysfunction and loss of synaptic connections within the retina. RGC axons atrophy by Wallerian degeneration and RGCs typically degenerate by apoptosis, with almost complete loss of RGCs noted at 2 weeks after crush injury.

In recent years, protective and regenerative activities of various inflammatory cell types and receptors have been identified in the optic nerve crush model, making this model useful for investigating signaling pathways that regulate the beneficial effects of inflammation. In this review, we describe the roles of inflammatory cells involved in detrimental and protective inflammation after optic nerve injury, review signaling pathways and interactions among inflammatory cell types, and highlight important directions for further research.

## Neuroinflammation After RGC Injury

The initial neuroinflammatory responses to neuronal injury stimulate intrinsic tissue reactions that function to limit further damage and restore tissue homeostasis. Damaged neurons release molecules such as damage-associated molecular patterns (DAMPs) and alarmins that activate inflammatory pathways in microglia, macrophages, and other inflammatory cell types, which act together to mediate the overall neuroinflammatory response. In many CNS injuries, such as multiple sclerosis and spinal cord injury, the initial inflammatory responses to neuronal damage are reparative. However, sustained or chronic inflammatory responses lead to the recruitment of additional inflammatory cells or alter their phenotypes and result in further neuronal loss [3–5].

Studies using animal models of RGC injury demonstrated that retinal inflammation contributes to RGC death and a progression of distinct immune cell types is observed at different timepoints after injury. Inflammatory responses occur at the site of injury in the optic nerve and within the retina adjacent to the RGC cell bodies (Fig. 1). Histological and functional evidence from retinal ischemic reperfusion, glaucoma, and optic nerve crush models demonstrated increased numbers of inflammatory cells after injury. Furthermore, reducing inflammation with anti-inflammatory drugs or genetic deletions of key inflammatory genes led to reduced RGC death. For example, the administration of anti-inflammatory agents such as tumor necrosis factor-alpha (TNFα) inhibitors [6], minocycline, and FasL-Fas signaling inhibitors [7] led to lower immune cell infiltration and reduced axonal degeneration and RGC death in rodent models of glaucoma [8]. Similarly, RGC survival improved in mice lacking interleukin 6 (IL-6) in a glutamate toxicity model [9], and mice with a genetic deletion of the innate immune receptor toll-like receptor 4 (TLR4) gene showed increased RGC survival after optic nerve crush (ONC) injury [10]. Therefore, inflammation-associated neurotoxicity on RGCs in these experimental models is consistent with the damaging effects of inflammation in other neurologic tissues such as the brain and spinal cord. Although it is useful to consider findings in one injury model as a starting point for generating hypotheses and identifying inflammatory mediators that may play roles in other injury types, it should be noted that extrapolating findings among different injuries may overlook differences in the initial inflammatory responses that may be specific to a particular injury model.

In contrast to pro-inflammatory neurotoxicity, there is a growing body of evidence indicating that some inflammatory cells and molecules have protective effects on RGCs and the optic nerve after injury (Table 1). For example, stimulation of intraocular inflammation using various approaches such as lens injury [11] or injection of zymosan [12] or crystallins [13] after optic nerve damage resulted in increased optic nerve regeneration and RGC survival. This regenerative inflammation in the retina and optic nerve is associated with specific subtypes of microglia, neutrophils, and certain secreted molecules, described in detail below. Therefore, characterizing inflammatory cell types and signaling pathways involved in neuroprotective inflammation is an essential step not only for understanding intrinsic biological responses to injury and disease but also for developing potential therapeutic strategies that control the migration and phenotype of inflammatory cells.

**Table 1:**
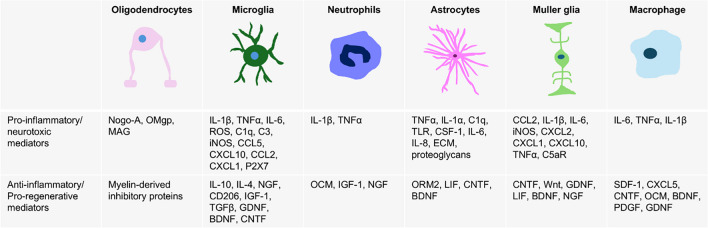
Molecular mediators of inflammation and axonal regeneration after RGC injury

## Inflammatory Cell Types in the Retina and Optic Nerve

### Microglia

#### Function

Microglia are the main effectors of the innate immune system in the CNS [14]. These indispensable cells are a type of mononuclear phagocyte that originates from the yolk sac, and they play essential roles during retinal development by phagocytosing excess RGCs and facilitating synaptic pruning. The main role of microglia in an adult retina is to respond to tissue damage and restore homeostasis, and their elongated cellular processes allow surveillance of the microenvironment and rapid reaction to pathologic conditions. Resident microglia are typically located in the nerve fiber layer (NFL) and the inner plexiform layer (IPL) and respond to injury to the retina and optic nerve. After an injury, microglia are activated, adopt an amoeboid morphology, and migrate to areas of damaged neurons. In mouse models of optic nerve damage, microglia migration and proliferation are prominent within 24 h after injury and reach maximum levels 2 days after injury before declining [15]. Microglia secrete inflammatory mediators that recruit other inflammatory cells and promote phagocytosis and clearance of dead cells and debris [16, 17]. Microglia infiltrating from the circulation [18] and optic nerve [19] also contribute to the injury response, although to a lesser extent.

Analysis of microglia transcriptomes using scRNAseq and other methods demonstrated substantial functional and phenotypic diversity [20]. Although the earlier classification of microglia into distinct “M1” or “M2” phenotype subtypes is not likely fully representative of microglial in vivo, these categories and their associated marker proteins are useful for describing potential microglial phenotypes. Classically activated pro-inflammatory microglia are often referred to as “M1-like” and express the marker protein inducible nitric oxide synthase (iNOS). These microglia cells secrete pro-inflammatory cytokines, proteases, nitric oxide, and superoxide and are associated with neurotoxicity and detrimental inflammatory functions [21]. In contrast, alternatively, activated myeloid cells are referred to as “M2-like” and express arginase 1 (Arg1) and secrete anti-inflammatory cytokines such as IL-10. These microglia mediate anti-inflammatory signaling, are associated with tissue-protective responses and reparative signaling [22], and are often increased by neuroprotective experimental treatments. For example, Arg1 + microglia were identified as potentially neuroprotective inflammatory cells in mouse retina after experimental treatments for optic nerve injury [23]. It remains to be determined whether Arg1 + microglia have beneficial roles in human retina.

#### Neurotoxic Microglia Phenotypes

Excessive or chronic microglial responses contribute to RGC death after injury or disease in glaucoma [24] and optic nerve injury [16]. Commonly identified microglial-derived cytokines, including IL-1β, IL-6, and TNF-α [17], as well as reactive oxygen species (ROS) [25], are neurotoxic and reduce RGC survival [26]. Furthermore, anti-inflammatory cytokines, such as IL-10 and IL-4, decline after injury [27]. Direct evidence for the toxic effects of cytokines on RGCs was demonstrated in primary RGC cultures in which TNF-α induced cell death [28] and anti-inflammatory IL-10 reduced RGC death [29]. Thus, alterations in tissue levels of microglial-secreted cytokines play a role in promoting damage to RGCs and axons following injury. Interestingly, differential survival of RGC subtypes after injury is well-established and is attributed to intrinsic properties within RGCs [30, 31]. However, whether microglia-induced neurotoxicity contributes to differential survival rates of these RGC subtypes has not been examined and should be a topic of future studies.

Another mechanism of microglial toxicity is through activation of the NLRP3 inflammasome. Analysis of microglia-mediated toxicity in a mouse model of traumatic optic neuropathy demonstrated that upregulated JNK/c-Jun signaling and NLRP3 inflammasome activation in retinal microglia resulted in axonal degeneration and RGC death [32]. Furthermore, mice with genetic loss of the NLRP3 inflammasome had delayed RGC loss, decreased axonal degeneration, and reduced caspase-1 and IL-1β levels [33]. The neuroprotective ketogenic diet was also associated with reduced microglia/macrophage in the retina and increased RGC survival in mice after ONC [34], and ketogenic-induced neuroprotection involves downregulating NLRP3 inflammasome-mediated pro-inflammatory responses [35]. Although the ketogenic diet is usually considered anti-inflammatory [36], additional evidence is required to determine whether reduced inflammation and inflammatory cell infiltration are involved in neuroprotection by the ketogenic diet.

Complement activation also contributes to microglia-induced RGC neurotoxicity. Complement protein C1qa is expressed in optic nerve microglia, and genetic deletion of C1qa reduced RGC death and optic nerve degeneration and prevented loss of RGC dendrites in the DBA/2J mouse model of glaucoma [37]. In a mouse optic nerve injury model, endogenous expression of complement proteins C3, C1q, and CR3 increased after injury, and deletion of any one of these complement proteins decreased RGC axonal growth [38]. These analyses provide strong support for the essential function of complement in regulating optic nerve regeneration.

#### Modifying Microglia Phenotypes Towards Neuroprotection

Suppressing detrimental microglial inflammatory responses was shown to protect RGCs in several injury models. For example, minocycline treatment of a mouse model of glaucoma led to decreased retinal microglia activation and improved RGC survival [39, 40]. In another study, inhibiting microglial aldose reductase, which mediates inflammatory responses, led to decreased IBA1-positive microglia/macrophage in the retina and optic nerve and improved RGC survival and function after ONC [41]. These studies are consistent with the findings in other CNS injury and disease models [4].

Although the neurotoxic activities of microglia are well-described, there is increasing evidence that microglia promote neuroprotection after various types of retinal injuries. Microglia have been shown to secrete growth factors and anti-inflammatory molecules that are neuroprotective after injury to the optic nerve [27, 42]. Additionally, in the NMDA-induced retinal excitotoxic injury mouse model, ablating microglia using PLX5622 led to decreased RGC survival [43]. Further analysis determined that exogenous IL-1β induced retinal microglia activation and improved RGC survival in damaged retinas, and IL-1β dependent neuroprotection was mediated by interleukin-1 receptor type 1 (IL-1R1) signaling in retinal astrocytes, indicating a potential protective microglia-astrocyte interaction [43]. Another study found that valproic acid applied to a retina explant axotomy model induced microglia to change their phenotype to the surveillance predominant M2-like microglia phenotype and subsequently reduced expression of pro-inflammatory genes, including CCL5, CXCL10, CCL2, CXCL1, and cystatin C [44]. Similarly, in the mouse ONC model, progesterone treatment led to fewer amoeboid-shaped microglia in the retina; reduced M1-like microglia phenotype markers including iNOS, TNF-α, and IL-6, and increased M2-like microglia phenotype markers including Arg1, CD206, and IL-4 [45], which was associated with increased RGC survival [45]. These in vivo studies provide important evidence for neuroprotective microglia activity in the retina in response to RGC and axonal injury.

The protective molecule gadolinium chloride also reduced retinal microglial proliferation after ONC and led to decreased M1-like microglial markers and increased M2-like markers, which were associated with increased RGC survival and axonal density [46]. In contrast, Wnt3a-induced RGC survival and axonal regeneration were associated with a twofold increase in microglia in the GCL, suggesting that Wnt3a increased the proliferation of microglia with neuroprotective phenotypes [47]. Minocycline, which increased RGC survival in rodent models [39, 40], also induced a phenotypic shift towards M2-like neuroprotective microglia and improved neuronal survival in a retinal degeneration model [48], indicating that minocycline likely improves RGC survival by promoting protective microglial phenotypes in addition to reducing pro-inflammatory microglia. Therefore, these consistent findings across multiple studies provide evidence for protective effects of retinal and optic nerve microglia after RGC and axonal injuries.

A mechanism of microglia-mediated RGC survival may involve the expression of protective molecules, either directly from microglia themselves or by stimulating growth factor secretion from Muller glia or other cell types. Microglia secrete various neuroprotective growth factors that promote neuronal survival in other CNS tissues and potentially induce RGC survival and regeneration after injury. These include insulin-like growth factor 1 (IGF-1) [49], transforming growth factor beta (TGFβ) [50], IL-6 [51], glial cell-derived neurotrophic factor (GDNF), brain-derived neurotrophic factor (BDNF), and ciliary neurotrophic factor (CNTF) [52]. These growth factors induce microglial and macrophage proliferation [53] and activate RGC pro-survival pathways. For example, CNTF promoted axonal regeneration and increased RGC survival in mice after ONC [54], and intraocular injection of IGF-1 increased RGC survival after optic nerve transection in mice [55]. Microglia also express complement receptor C3, and complement protein C3a promotes the expression of nerve growth factor (NGF) in cultured human microglia, suggesting that complement indirectly induces microglia-mediated neuroprotection by elevating pro-survival neurotrophins [42]. A protective function for C3 on RGC survival was shown by genetic deletion of C3 in the DBA/2J glaucoma model [56], but the opposite effect was shown in another study [57], indicating the complex effects of complement signaling. Furthermore, although these growth factors are protective when injected or over-expressed, it has not yet been shown whether they reach high enough levels to induce protection when secreted by microglia in vivo.

The extent of microglial involvement in RGC survival and axonal regeneration in the optic nerve varies across studies. As described above, retinal microglia have generally been shown to contribute to some extent to damage and regeneration responses after optic nerve injury by playing protective or toxic roles. An interesting exception is a study by Hilla et al. [58], which showed that depleting microglia using the colony-stimulating factor 1 receptor (CSF1R) inhibitor PLX5622 did not change lens injury-induced axonal regeneration in an optic nerve injury model, although did impair phagocytosis of apoptotic RGCs. Although the reason for the lack of effect of PLX5633 was not determined, it was shown in other studies that the timing and location of microglia activation alters their responses to damage, and the roles of microglia after optic nerve injury depend on their site of action. Siddiqui et al. [59] demonstrated that microglia stimulated into a pro-inflammatory phenotype had an early neuroprotective effect at the optic nerve but promoted RGC death when acting on the RGC soma, whereas anti-inflammatory microglia were neuroprotective at the RGC soma but not at the optic nerve. Therefore, studies using longitudinal transcriptional profiling of the retina and optic nerve will be important for defining altered microglial responses after injury and during axonal regeneration. As an example, analysis of microglia cell populations after traumatic brain injury demonstrated pro- and anti-inflammatory gene expression changes in a time-dependent manner, rather than a general shift towards distinct pro-inflammatory or anti-inflammatory phenotypes [60, 61]. Additional characterization of microglia phenotypes in the retina is needed, including defining temporal and spatial patterns of microglia migration and polarization, and determining whether RGC metabolic changes and activity could influence microglia responses.

A promising area of research for developing new treatments aimed at preventing RGC death is reducing the neurotoxic effects of microglia while enhancing their neuroprotective functions. Several molecules and signaling pathways are known to dampen neurotoxic microglial responses and promote the secretion of neuroprotective and anti-inflammatory molecules [48, 62], which suggests potential approaches for modulating microglial responses for therapeutic use. For example, studies in cultured cells and animal models demonstrated that IL-4 [15], IL-13 [63], and myeloid differentiation factor 88 (MyD88) inhibitors [64] change the proportion of neuroprotective and neurotoxic microglial phenotypes. Additionally, astrocytes secrete molecules that modulate microglia activity and migration in culture, such as Orm2 [65], which potentially also occur in vivo to resolve inflammation. Further research is needed to fully characterize the signaling proteins and cell types that contribute to tissue recovery, in order to allow precise control of protective and detrimental inflammatory responses.

### Macrophages

#### Functions and Comparisons to Microglia

Resident and infiltrating macrophages differentiate from blood-derived monocytes and respond to retinal disease and injury [14]. Retinal microglia and macrophages have overlapping functions and express similar receptors but often have distinct roles in retinal homeostasis and disease [14]. Macrophages enter the damaged retina 2 to 3 days after injury and show peak activation approximately 1 week after injury, with sustained phagocytotic activity up to 6 weeks after injury. In contrast, microglia show earlier activation, and their phagocytotic activity peaks 1 to 3 days after injury and diminishes afterward [66]. Peripheral macrophages infiltrate the axonal injury site after ONC but not the retina [58] unless inflammation is directly stimulated [67]. It is important to note that many studies do not distinguish “true” yolk-sac-derived microglia from other microglial- and macrophage-like cells. Commonly used protein markers such as ionized calcium-binding adaptor molecules (IBA1) detect both microglia and macrophages. Similarly, CD86 is often used as a marker for macrophages, although it was detected in several other cell types, including microglia [68]. While IBA1, CD86, and similar markers cannot distinguish between the two cell types, these markers are appropriate for measuring major reactive phagocytic inflammatory cells after injury, which includes microglia and macrophages, and for quantifying neuroinflammation and cell migration within the tissue in response to damage. Microglia can be distinguished from macrophages by measuring levels of CD45 and CD11 using flow cytometry [62, 69, 70], although this analysis requires tissue dissociation and does not allow quantification of cells within specific retinal layers. A sophisticated approach to track and quantify macrophage and microglia separately is to use mouse transgenic lines that express a reporter gene in distinct monocytes, which can be combined with marker antibodies and even cell type-specific depletions. Therefore, it is important to consider the detection methods and timepoints after injury when evaluating the roles of macrophages in optic nerve regeneration.

#### Pro-Regenerative Activity

A study by Leon et al. (2000) reported that lens damage after optic nerve injury in a rat model induced infiltration of CD68-positive macrophages into the eye, which was associated with improved RGC survival and axonal regeneration [11]. In a subsequent analysis, zymosan injection combined with a peripheral nerve graft after optic nerve injury also resulted in macrophage accumulation in the vitreous and the retinal GCL and was associated with increased RGC survival [67]. Furthermore, monocyte-derived retinal macrophages that were stimulated by injury in the glutamate toxicity model led to downregulated proinflammatory cytokines, upregulated anti-inflammatory cytokines, and neuroprotective pathways, leading to increased RGC survival. Removal of these macrophages using anti-C–C chemokine receptor type 2 (CCR2) antibodies reduced RGC survival [27]. In another study, transplanted macrophages promoted axonal regrowth of transected optic nerve in rats, and the absence of intrinsic axonal growth was hypothesized to be caused by the lack of sufficient macrophage activation [71]. Furthermore, conditioned media from zymosan-activated macrophages induced neurite growth in primary RGC cultures, providing further support for a pro-regenerative role for macrophages [72].

Macrophages in the optic nerve may promote axonal regrowth by removing cellular debris such as myelin and by regulating microglial activity. A recent study demonstrated that inflammation-induced optic nerve regeneration was associated with increased microglia/macrophage phagocytosis and myelin removal [73]. Macrophages also promote neuroprotection by regulating pro-inflammatory microglia. Using a bilaminar co-culture system, macrophages were shown to suppress microglia-mediated phagocytosis and decrease pro-inflammatory cytokines IL-1β, TNF, and IL-6, whereas microglia increased macrophage-mediated phagocytosis [74]. This finding suggests that the delayed appearance of macrophages may serve to modulate the earlier activated microglial response. In vivo interactions between the cell types were shown in a spinal cord injury model, in which blocking macrophage infiltration in CCR2 knockout mice resulted in prolonged microglia activation and decreased locomotor recovery [74], but studies have not yet shown similar interactions in retina or nerve.

Another potential macrophage-mediated mechanism of neuroprotection is the expression of neuroprotective chemokines and neurotrophic factors. For example, the chemokine stromal cell-derived factor 1 (SDF-1) is expressed by infiltrating macrophages in the retina and inducing RGC survival and axonal regeneration after optic nerve injury [75]. The chemokine C-X-C motif chemokine ligand 5 (CXCL5) is upregulated after optic nerve injury and leads to the proliferation of CD68 + macrophages and increased RGC survival [76]. Furthermore, CNTF, a neurotrophic factor known to induce axonal regeneration and RGC survival, was also found to be a chemoattractant of blood-derived macrophages [77], suggesting potential signaling pathways for macrophage-mediated RGC protection.

Oncomodulin (OCM) is a calcium-binding protein secreted by retinal macrophages and neutrophils in response to intraocular inflammation that regulates macrophage pro-regenerative functions [78]. OCM is expressed in CD68-positive infiltrating macrophages in aqueous and vitreous and it mediates axonal growth and RGC survival in association with lens injury-induced intraocular inflammation [79]. Intraocular injection of OCM-inhibiting peptides [79] or anti-OCM antibodies [80] at the time of lens injury led to decreased inflammation-induced optic nerve regeneration, although neither led to improved RGC survival. Therefore, intriguing evidence demonstrates that OCM is an example of an inflammatory protein with pro-regenerative effects secreted by macrophages in the retina.

#### Neurotoxic Activity

An ex vivo study using rat retinas co-cultured with peritoneal macrophages showed decreased RGC survival and axonal growth, which worsened when the peritoneal macrophages were first activated by zymosan [81]. Furthermore, hematogenous macrophages promote fibrotic scar formation after optic nerve injury in mice, and macrophage depletion reduced scar formation although did not rescue RGCs [82]. In summary, the majority of evidence suggests that macrophages primarily promote pro-regenerative inflammatory processes. Although some detrimental activities have also been reported, these findings require confirmation in additional studies.

### Muller Glia

#### Functions

Muller glia are a type of radial glial cell that play essential roles in retinal homeostasis and function, and they respond to retinal injury and communicate with inflammatory cells [83]. Muller glia express inflammatory receptors such as TLR4 [84] and secrete numerous cytokines, chemokines, and neurotrophic factors [83]. In rodent models of RGC damage, Muller glia were shown to influence RGC survival and optic nerve regeneration by secreting neuroprotective and pro-regenerative molecules in response to Wnt ligands [47, 85] and CNTF [86]. Muller glia activation contributes to gliosis, which may be detrimental to tissue recovery or may wall off damaged neurons and promote tissue restoration [87, 88]. Additionally, recent studies indicate that Muller glia-regulated inflammatory pathways play direct roles in pro- and anti-regenerative responses, as described below.

#### Muller Glia-Microglia Cross-Talk

Muller glia communicate with retinal microglia through various signaling pathways, and this cross-talk plays roles in inflammation, neuronal survival, and axonal regeneration. For example, in an induced glaucoma mouse model, Muller glia led to increased microglia activation through ATP/P2X7 signaling. Furthermore, a P2X7R agonist promoted an M1-like phenotype in microglia cultures, whereas inhibiting P2X7R or Muller glia activation resulted in increased RGC survival [89]*.* Similarly, zymosan-induced inflammation in a zebrafish ONC model led to immune cell infiltration, Muller glia proliferation, gliotic responses, and axonal regeneration. By comparison, zebrafish mutants that lacked microglia showed modified Muller glia gliosis and increased axonal regeneration after optic nerve injury, suggesting interactions between Muller glia and microglia regulate axonal regrowth [90]. Additionally, microglia-derived CNTF stimulated neurotrophic factor secretion from Muller glia in a mouse model of photoreceptor degeneration, implying a similar effect may occur after RGC injury [52]. In a rat model of light damage, which primarily injures photoreceptors, reducing CCL2 in Muller glia using siRNA led to decreased microglia recruitment and increased photoreceptor survival, suggesting that Muller glial-secreted CCL2 contributes to microglia migration during the retinal damage response [91]. Therefore, a large body of evidence supports the role of Muller glia in the inflammatory reactions to retinal injury.

Another major line of research investigates how microglia regulate Muller glia proliferation and reprogramming into new neurons in injury models [92, 93]. Studies in zebrafish, chick, and mammalian retinas demonstrated that Muller glia are a source of new neurons after injury, and the extent of neuronal replacement varies across species [94]. Reprogramming of Muller glia into RGCs is far less efficient in mammals than other species. Therefore, studies comparing highly regenerative zebrafish Muller glia to poorly regenerative mammalian glia will provide insight into inflammatory and genetic pathways that influence microglia-Muller glia interactions and regulate progenitor cell reprogramming into neurons during different injury conditions.

Muller glia-microglia co-cultures have been used to explore the molecular mechanisms of their interactions. The TNFR1-NFκB signaling pathway mediates Muller glia-microglia cross-talk in which microglia-secreted TNF-α induced Muller glia activation in a TNF receptor 1 (TNFR1)-dependent manner [95]. Furthermore, inhibiting TNFR1 reduced NFκB-mediated activation of Muller glia by microglia in co-cultures [95]. In another study, Muller glia co-cultured with activated microglia showed reduced expression of the Muller glia gliosis markers glutamate aspartate transporter (GLAST) and vimentin, suggesting that microglia may dampen gliotic responses and alter intrinsic tissue restoration processes [96]. Additionally, Muller glia co-cultured with activated microglia, but not inactivated microglia, showed elevated expression of inflammatory factors IL-1β, IL-6, and iNOS and upregulated growth factors GDNF and leukemia inhibitory factor (LIF) [96]. In the same study, conditioned media from Muller glia-microglia co-cultures induced microglia proliferation and increased cytokine expression [96]. Therefore, bi-directional interactions between microglia and Muller glia may induce neuroprotective signaling processes during neuroinflammatory damage responses, with the caveat that extrapolation from in vitro studies to in vivo should be done cautiously.

Muller glia responses to retinal injury include secretion of neuroprotective neurotrophic factors such as BDNF, nerve growth factor (NGF), and GDNF, among others [97, 98]. The combination of microglial- and Muller-glia-derived secretion of these protective proteins may increase the total levels in the retina, potentially restore homeostasis, and enhance RGC survival and axonal regeneration [99]*.*

#### Neuroinflammatory and Neuroprotective Activity

Rodent Muller glia express Toll-like receptors (TLRs) along with other inflammatory receptors. Stimulation of TLR2 and TLR4 signaling leads to expression of the cytokine IL-6, chemokine macrophage inflammatory protein 2 (MIP-2)/CXCL2 [100], and nuclear factor kappa B (NFκB) signaling activation [84]. Analysis of a human Muller glia cell line stimulated with the TLR4 activator LPS using mass spectrometric proteomics profiling showed increased expression of proteins related to antigen-presenting cells, raising the question of whether Muller glia acts as an atypical antigen presenting cell (APC) during neuroinflammation [101]. However, this possibility has not been explored in optic nerve injury models.

Muller glia secrete and respond to various inflammatory mediators [102]. For example, cultured Muller glia treated with IL-1β induced the expression of multiple cytokines and chemokines, including IL-6, CCL2, CXCL1, and CXCL10 [103, 104]. The IL-6 family member LIF and its receptors are expressed by Muller glia [105], and LIF induces Muller glia activation and gliosis after optic nerve injury. LIF knock-out mice show reduced Muller glia activation and suppressed gliosis after ONC [106]. Furthermore, LIF promotes axonal growth in cultured RGCs and is a mediator of inflammation-induced optic nerve regeneration [107]. In contrast, TNFα secreted by Muller glia was implicated in RGC death after excitotoxic injury [108]. Therefore, evidence in vitro and in vivo supports Muller glia as an active inflammatory cell type.

Wnt signaling pathways regulate essential processes in normal and diseased tissue, including injured retina [109]. Wnt signaling in Muller glia induced RGC neurite growth, caused secretion of regeneration and survival factors in cultured RGCs [85], promoted axonal regeneration, and enhanced RGC function after ONC in vivo [47, 110]. Wnt signaling is influenced by inflammatory molecules and plays a role in the regulation of inflammation. For example, TLR4 signaling decreased Wnt signaling in Muller glia-photoreceptor co-cultures [84], and Wnt signaling inhibition in a mouse model of encephalopathy was required for pro-inflammatory microglia activation [111]. Similarly, the Wnt/β-catenin activator Norrin, which is expressed in Muller glia [112], increased RGC survival in an RGC toxicity model [113]. Therefore, Muller glia contributes to axonal regeneration through Wnt signaling and other inflammatory and non-inflammatory pathways.

Muller glia may also modulate inflammatory responses to injury through the expression of complement system proteins, including C1q [114], C1, C3, factor B (C2), and C1 inhibitor [115]. Complement receptor C5aR is active on cultured Muller glia and leads to secretion of IL-6 and vascular endothelial growth factor (VEGF) [116], although it is not known whether complement factors are differentially expressed by Muller glia after optic nerve injury. While current evidence suggests that Muller glia regulate the complement system and gliosis by secreting multiple cytokines and chemokines, the precise role of Muller glia in neuroinflammation after optic nerve damage requires further investigation. Therefore, a promising area of future research is defining the contribution of Muller glia to pro-regenerative inflammation.

### Astrocytes

#### Functions

Astrocytes are a type of glial cell located in the retinal NFL and GCL that play multiple important roles within the retina, including retinal development, metabolic homeostasis, and response to injury [117]. During development, astrocytes regulate retinal vessels and RGC synapse formation [118] and contribute to immune privilege in the eye by maintaining the blood-retinal barrier [119]. Astrocytes in the retina, optic nerve head, and optic nerve react to RGC damage and inflammation [120]. These astrocytes adopt different reactive states with neurotoxic or neuroprotective phenotypes that are designated as A1 and A2, respectively [121, 122].

#### Neurotoxic Astrocyte Activity

When cultured with RGCs, forebrain-derived A1 astrocytes reduced synapse maintenance, decreased synaptic firing, and reduced RGC survival [121]. However, injection of astrocyte-conditioned media into uninjured mouse retina did not alter RGC survival, indicating that factors secreted by astrocytes are not sufficient to promote RGC death [120].

Astrocyte-microglia interactions are involved in detrimental damage responses in certain situations. For example, astrocyte gap junctions, which are vital to maintaining neuronal homeostasis, can be suppressed by pro-inflammatory cytokines secreted by microglia in culture [123]. Neurotoxic A1 astrocytes in the retina are activated by microglia via TNF-α, IL-1α, and C1q, which leads to neuronal death [121]. In a mouse model of ONC, genetic deletion of TNF-α, IL-1α, and C1q prevented a phenotypic shift towards neurotoxic retinal astrocytes and led to increased RGC survival, which provides evidence that these cytokines play a detrimental role in astrocyte function and neuronal survival [121]. Microglia also contribute to astrocyte recruitment to the optic nerve lesion site, as demonstrated using PLX5622 treatment to deplete microglia after optic nerve injury which resulted in delayed recruitment of astrocytes to the injury site [58]. Furthermore, molecular cross-talk may exist between astrocytes and Muller glia because they are physically adjacent within the retina, and transport of metabolites and lipid molecules could occur between these glial cell types and RGCs [87], although the effect on inflammation and axonal regeneration is unknown. Further studies are needed to determine whether astrocytes interact with Muller glia to regulate the effect of inflammatory pathways on RGC survival and axonal regrowth.

A major signaling pathway that mediates astrocytic involvement in the inflammatory responses to injury is toll-like receptors (TLR), which play central roles in pathogen recognition and sterile activation of the immune response [124]. TLRs are expressed on astrocytes and other immune cells, as well as neurons and glia [84, 125]. Several studies demonstrated that TLR activation contributes to RGC death. For example, mice with a global knockout of the TLR4 gene showed reduced RGC death after optic nerve injury [10], and inhibiting TLR4 in the retina after ONC led to increased RGC survival, decreased activation of astrocytes and reduced NFκB expression [126]. Similarly, genetic loss of TLR9 signaling led to improved RGC survival after optic nerve injury [127]. TLRs are expressed in astrocytes and microglia in glaucomatous tissue from the human retina, suggesting a potential role in the pathophysiology of RGC death in glaucoma [128].

#### Beneficial Role for Astrocytes

A2 reactive astrocytes play neuroprotective roles in the inflammatory response to injury and improve RGC survival [121]. The neuroprotective astrocyte phenotype is characterized by the upregulation of neurotrophins, including LIF [107], BDNF [129], and CNTF [86, 107], which are protective molecules that increase RGC survival. Analysis of a mouse model of glaucoma demonstrated increased expression of A2 astrocyte genetic markers in the optic nerve head, whereas microglia depletion promoted a shift from A2 astrocytes towards the neurotoxic A1 phenotype and was associated with decreased RGC survival [125]. This finding suggests that the proportion of A2 astrocytes may depend on the functions of activated microglia [130]. However, less is known about the roles of astrocytes with the A2 phenotype in the retina and optic nerve compared to the A1 phenotype, and further study will be needed to determine their contributions to regenerative processes after injury.

Reactive astrogliosis is influenced by many inflammatory signaling pathways. One example is the transcription factor STAT3, which may promote neuroprotective inflammation by inducing a shift of astrocytes towards the A2 phenotype [131]. STAT3 knockdown in a mouse model of ONC led to reduced astrocyte activation in the optic nerve, increased RGC death, and loss of visual function [132]. In contrast, NFκB, another critical transcription factor that mediates inflammatory responses, activates astrocytes towards the A1 phenotype when analyzed in the brain and spinal cord [133, 134]. Blocking astrocyte-induced NFκB signaling in the optic nerve in an optic neuritis model led to decreased pro-inflammatory responses, reduced NOS2 levels, and increased RGC survival [135].

CNS regeneration after injury is generally inhibited by astrocyte-regulated glial scars. Reactive astrogliosis induces inflammatory responses, including secretion of inflammatory mediators TNF-α [136], colony-stimulating factor 1 (CSF-1) [137], IL-6, and IL-8 [138]. Reactive astrocytes also produce extracellular matrix proteins and other growth-inhibitory molecules such as proteoglycans that contribute to axonal growth inhibition [139]. For example, α-crystallin-induced axonal regeneration was associated with decreased expression of neural-glial antigen 2 (NG2) proteoglycan and neurocan in astrocytes in the retina and optic nerve in a mouse ONC injury model [140]. Astrocyte secretion of chondroitin sulfate proteoglycans (CSPG) after injury was shown to be a barrier to axonal regeneration [140]. Chick embryonic stem cells implanted into transected rat optic nerve caused matrix metalloprotease activation in astrocytes and subsequent CSPG degradation, which permitted axons to regenerate through the optic nerve to the thalamus [141]. The astrocyte-induced glial scar may also suppress pro-regenerative inflammatory cell infiltration into the damaged nerve, which would reduce regeneration [142]. Further study is needed to define how astrocytes interact with other immune cells and Muller glia, and identify which secreted proteins contribute to the glial scar and regulate RGC survival and optic nerve regeneration.

### Neutrophils

There is increasing evidence that neutrophils play important roles in the acute inflammatory response to optic nerve injury and regulation of axonal regeneration. Neutrophils are often one of the first immune cell types to migrate to the injury site, and possibly provide the foundation for subsequent tissue regeneration [143]. Although neutrophils are pro-inflammatory and often detrimental, neutrophil depletion reduced axonal regeneration in a mouse ONC model, indicating that specific neutrophil subtypes may be necessary for restoring neuronal homeostasis [80]. Neutrophils were shown to express high levels of the calcium-binding protein OCM after zymosan-induced inflammation, and depletion of neutrophils in the retina using Ly6G-specific monoclonal antibody caused decreased retinal OCM and reduced axonal regeneration [80]. In a direct test of the role of neutrophils after ONC, adoptive transfer of a subset of mouse neutrophils that were harvested 3 days after zymosan-induced inflammation and injected into mouse eyes after ONC injury resulted in increased RGC survival and significant axon regeneration [23]. Furthermore, neutrophil activation was shown to be required for CNTF-induced axonal regrowth [144]. Further research is needed to understand the contribution of neutrophils to optic nerve regeneration, including whether neutrophils cooperate with microglia, dendritic cells, and other cell types [23], and to identify their secreted neuroprotective and regenerative factors.

### Oligodendrocytes

Myelination of axons by oligodendrocytes enables efficient electrical conduction of neuronal signals. Optic nerve injury leads to loss of oligodendrocytes, which impairs clearance of myelin debris and reduces remyelination of regrowing axons. In a recent study by Xing et al. [145], most oligodendrocytes in the optic nerve were observed to be lost 1 day following ONC injury, after which repopulation by newly born oligodendrocytes occurred 2 weeks after injury. These new cells were localized to the glial scar [145]. Oligodendrocyte progenitor cells (OPCs) proliferate and form mature oligodendrocytes after injury and this proliferation typically depends on microenvironment factors including trophic factors and electrical activity in adjacent neurons. Interactions between OPCs and microglia and astrocytes also influence OPC differentiation, maturation, and myelin production [146]. However, axons fail to be remyelinated following optic nerve injury due to OPC daughter oligodendrocytes lacking myelination capacity [147].

Several studies demonstrated that myelin-associated proteins and myelin debris inhibit regrowing axons. Furthermore, the presence of newly formed oligodendrocytes that arise after injury within the glial scar reduces the growth of new axons, although the precise mechanisms are unknown. These post-injury born oligodendrocytes express myelin-associated axon growth inhibitors, including NogoA, OMgp, and MAG, which may inhibit axonal regeneration [145]*.* Additionally, increased axonal growth was demonstrated using demyelination treatment and pharmacologic agents that reduced the interaction of regenerating axons with post-injury born oligodendrocytes in the glial scar [145].

Appropriate remyelination of new axons is essential for regaining optic nerve function. The proliferation of OPCs, differentiation of OPCs to oligodendrocytes, and myelination capacity of newly produced oligodendrocytes were studied in several injury models and yielded differing conclusions regarding the ability of oligodendrocytes present at the injury site to remyelinate axons following injury. It was recently shown that axons fail to be remyelinated following optic nerve injury due to OPC daughter oligodendrocytes lacking myelination capacity and that manipulating OPC genetic pathways could promote remyelination [147]. Promoting myelination using a GPR17 inhibitor combined with microglia depletion increased myelination of regenerating axons after optic nerve injury in a CNTF-induced axonal regrowth model [147]. Further research aimed at investigating myelination capacity at different time points following axonal injury is needed to fully understand the contribution of OPCs to optic nerve functional regeneration.

Other cell types also contribute to oligodendrocyte-mediated remyelination after optic nerve injury. Analysis of various CNS tissues after injury has found mostly positive functions for microglia on OPC proliferation or maturation. For example, in a spinal cord injury model, pro-inflammatory signaling from microglia and macrophages via MyD88 was required for clearance of myelin debris and for initiating oligodendrocyte repopulation and remyelination [148]. Other studies showed that initial microglial responses promote phagocytosis of myelin debris, followed by inflammation resolution and a shift of microglia to an M2-like phenotype [149]. Miron et al. demonstrated using a mouse model of multiple sclerosis (MS) that the shift to the M2 microglia/macrophage phenotype following demyelination injury occurs at the beginning of remyelination of regenerating axons and is required for efficient remyelination [150]. Similarly, in the mouse cuprizone model of MS, microglia recruit OPCs to the lesion site and secrete molecules that are proposed to support OPC function and remyelination, thereby promoting tissue regeneration and homeostasis through beneficial oligodendrocyte interactions [151]. However, in the optic nerve crush model, experiments using microglia depletion demonstrated that microglia promoted injury-induced OPC proliferation but prevented OPC maturation, indicating that the role of microglia in oligodendrocyte function is complex [147]. Further studies are needed to fully understand the role of microglia and other inflammatory cells in OPC proliferation and differentiation after ONC injury.

## Conclusions and Future Research Directions

New technologies, such as spatial transcriptomics, metabolomics, and other multi-omics analyses, as well as cell type-specific transgenic and knock-out mouse lines, are being used to address essential questions. As mentioned above, longitudinal transcriptional profiling of the retina and optic nerve could be used to define the progression of inflammatory cell responses after acute and chronic injury and to identify pro-regenerative inflammatory signaling pathways. Important questions for further investigation are defining the timing and phenotypes of cells that migrate to the injury site, whether interactions among inflammatory cells and other cell types, such as neurons and vascular cells, modify their phenotypes and damage responses, and how degenerating RGCs and cellular debris influence neuroinflammation activation and function. Furthermore, promising areas of future research could include investigating how environmental modifications, such as light stimuli and circadian rhythm, influence inflammatory cell responses to optic nerve injury. Additionally, because microglia and astrocytes are essential to RGC maturation and synapse refinement, it is important to determine whether the developmental functions of microglia or other inflammatory cells also contribute to injury outcomes. Finally, defining differences and similarities among inflammatory cell functions in animal models and humans will be essential, and using humanized transgenic mouse lines will allow the characterization of immune responses in appropriate contexts.

As described above, multiple inflammatory cell types and subtypes of those cells play key roles in RGC death and survival after injury. While there is a greater body of evidence supporting the contributions of microglia and macrophages in the inflammatory responses to RGC injury, a side-by-side comparison among these and other cell types has not been performed. Therefore, the relative importance of different inflammatory cell types following injury is not yet known and should be the focus of future studies.

The roles of inflammation in RGC survival and optic nerve regeneration after injury are complex, and both neurotoxic effects and neuroprotective effects of the inflammatory responses to optic nerve injury have been demonstrated. Low levels of inflammation immediately after damage can be beneficial to homeostasis restoration, protection, and regeneration, whereas chronic inflammation from over-activation or polarization to neurotoxic microglia and astrocytes has negative consequences on RGC survival and axonal regeneration [21]. Therefore, experimental strategies that selectively block neurotoxic inflammatory signaling and enhance protective and pro-regenerative inflammatory signaling pathways are predicted to be more beneficial to injured neurons than eliminating inflammation entirely. Many cell types, including microglia, Muller glia, astrocytes, neutrophils, and others, play essential roles in mediating both the protective and neurotoxic aspects of inflammation. Studies have also shown that targeting certain pathways related to inflammation, such as OCM, STAT3, Wnt, and others, can promote optic nerve axonal regeneration in animal models. Future therapeutic options for optic nerve injury and degeneration may involve targeting certain neuroprotective pathways and inhibiting neurotoxic inflammatory pathways in combination with guiding axons to their targets in the brain. Continued efforts to define the cells and signaling proteins involved will hopefully lead to the development of therapies modulating the inflammatory response to treat optic nerve injuries and pathologies.

## Data Availability

No datasets were generated or analysed during the current study.

## Abbreviations

*Arg1*: Arginase 1
*BDNF*: Brain-derived neurotrophic factor
*CCL2*: C-C motif chemokine ligand 2
*CCL5*: C-C motif chemokine ligand 5
*CCR2*: C-C chemokine receptor type 2
*CNS*: Central nervous system
*CNTF*: Ciliary neurotrophic factor
*CR3*: Complement receptor 3
*CSF-1*: Colony stimulating factor 1
*CSF1R*: Colony stimulating factor 1 receptor
*CSPG*: Chondroitin sulfate proteoglycans
*CXCL1*: CXC motif chemokine ligand 1
*CXCL10*: CXC motif chemokine ligand 10
*CXCL5*: CXC motif chemokine ligand 5
*DAMPs*: Damage associated molecular patterns
*GCL*: Ganglion cell layer
*GDNF*: Glial cell-derived neurotrophic factor
*GLAST*: Glutamate aspartate transporter
*IBA1*: Ionized calcium-binding adaptor molecule 1
*IGF-1*: Insulin-like growth factor 1
*IL-1R1*: Interleukin-1 receptor type 1
*iNOS*: Inducible nitric oxide synthase
*IPL*: Inner plexiform layer
*LIF*: Leukemia inhibitory factor
*MIP-2*: Macrophage inflammatory protein-2
*MyD88*: Myeloid differentiation factor 88
*NFkB*: Nuclear factor kappa B
*NFL*: Nerve fiber layer
*NG2*: Neural-glial antigen 2
*NGF*: Nerve growth factor
*NMDA*: *N*-Methyl-d-aspartate
*NLRP3*: NOD-like receptor family pyrin domain-containing 3
*OCM*: Oncomodulin
*ONC*: Optic nerve crush
*RGC*: Retinal ganglion cell
*SDF-1*: Stromal cell-derived factor 1
*STAT3*: Signal transducer and activator of transcription factor 3
*TGFβ*: Transforming growth factor beta
*TLR*: Toll-like receptor
*TNFR1*: Tumor necrosis factor receptor 1
*TNFα*: Tumor necrosis factor-alpha
*VEGF*: Vascular endothelial growth factor

## References

[1] Schwartz M (2004) Optic nerve crush: protection and regeneration. Brain Res Bull 62(6):467–471. 10.1016/S0361-9230(03)00076-5 15036559 10.1016/S0361-9230(03)00076-5

[2] Berkelaar M, Clarke DB, Wang YC, Bray GM et al (1994) Axotomy results in delayed death and apoptosis of retinal ganglion cells in adult rats. J Neurosci 14(7):4368–4374 8027784 10.1523/JNEUROSCI.14-07-04368.1994 PMC6577016

[3] Geloso MC, Corvino V, Marchese E, Serrano A et al (2017) The dual role of microglia in ALS: mechanisms and therapeutic approaches. Front Aging Neurosci 9:242. 10.3389/fnagi.2017.00242 28790913 10.3389/fnagi.2017.00242 PMC5524666

[4] Voet S, Prinz M, van Loo G (2019) Microglia in central nervous system inflammation and multiple sclerosis pathology. Trends Mol Med 25(2):112–123. 10.1016/j.molmed.2018.11.005 30578090 10.1016/j.molmed.2018.11.005

[5] David S, Kroner A (2011) Repertoire of microglial and macrophage responses after spinal cord injury. Nat Rev Neurosci 12(7):388–399. 10.1038/nrn3053 21673720 10.1038/nrn3053

[6] Nakazawa T, Nakazawa C, Matsubara A, Noda K et al (2006) Tumor necrosis factor-alpha mediates oligodendrocyte death and delayed retinal ganglion cell loss in a mouse model of glaucoma. J Neurosci 26(49):12633–12641. 10.1523/JNEUROSCI.2801-06.2006 17151265 10.1523/JNEUROSCI.2801-06.2006 PMC6674838

[7] Krishnan A, Kocab AJ, Zacks DN, Marshak-Rothstein A et al (2019) A small peptide antagonist of the Fas receptor inhibits neuroinflammation and prevents axon degeneration and retinal ganglion cell death in an inducible mouse model of glaucoma. J Neuroinflammation 16(1):184. 10.1186/s12974-019-1576-3 31570110 10.1186/s12974-019-1576-3 PMC6767653

[8] Bordone MP, Gonzalez Fleitas MF, Pasquini LA, Bosco A et al (2017) Involvement of microglia in early axoglial alterations of the optic nerve induced by experimental glaucoma. J Neurochem 142(2):323–337. 10.1111/jnc.14070 28498493 10.1111/jnc.14070

[9] Fisher J, Mizrahi T, Schori H, Yoles E et al (2001) Increased post-traumatic survival of neurons in IL-6-knockout mice on a background of EAE susceptibility. J Neuroimmunol 119(1):1–9. 10.1016/s0165-5728(01)00342-3 11525794 10.1016/s0165-5728(01)00342-3

[10] Morzaev D, Nicholson JD, Caspi T, Weiss S et al (2015) Toll-like receptor-4 knockout mice are more resistant to optic nerve crush damage than wild-type mice. Clin Exp Ophthalmol 43(7):655–665. 10.1111/ceo.12521 25752496 10.1111/ceo.12521

[11] Leon S, Yin Y, Nguyen J, Irwin N et al (2000) Lens injury stimulates axon regeneration in the mature rat optic nerve. J Neurosci 20(12):4615–4626. 10.1523/JNEUROSCI.20-12-04615.2000 10844031 10.1523/JNEUROSCI.20-12-04615.2000 PMC6772462

[12] Lorber B, Berry M, Logan A (2005) Lens injury stimulates adult mouse retinal ganglion cell axon regeneration via both macrophage- and lens-derived factors. Eur J Neurosci 21(7):2029–2034. 10.1111/j.1460-9568.2005.04034.x 15869497 10.1111/j.1460-9568.2005.04034.x

[13] Fischer D, Hauk TG, Muller A, Thanos S (2008) Crystallins of the beta/gamma-superfamily mimic the effects of lens injury and promote axon regeneration. Mol Cell Neurosci 37(3):471–479. 10.1016/j.mcn.2007.11.002 18178099 10.1016/j.mcn.2007.11.002

[14] Yu C, Roubeix C, Sennlaub F, Saban DR (2020) Microglia versus monocytes: distinct roles in degenerative diseases of the retina. Trends Neurosci 43(6):433–449. 10.1016/j.tins.2020.03.012 32459994 10.1016/j.tins.2020.03.012 PMC7556353

[15] Wohl SG, Schmeer CW, Witte OW, Isenmann S (2010) Proliferative response of microglia and macrophages in the adult mouse eye after optic nerve lesion. Invest Ophthalmol Vis Sci 51(5):2686–2696. 10.1167/iovs.09-4537 20007834 10.1167/iovs.09-4537

[16] Au NPB, Ma CHE (2022) Neuroinflammation, microglia and implications for retinal ganglion cell survival and axon regeneration in traumatic optic neuropathy. Front Immunol 13:860070. 10.3389/fimmu.2022.860070 35309305 10.3389/fimmu.2022.860070 PMC8931466

[17] Fan W, Huang W, Chen J, Li N et al (2022) Retinal microglia: functions and diseases. Immunology 166(3):268–286. 10.1111/imm.13479 35403700 10.1111/imm.13479

[18] Yuan TF, Liang YX, Peng B, Lin B et al (2015) Local proliferation is the main source of rod microglia after optic nerve transection. Sci Rep 5:10788. 10.1038/srep10788 26035780 10.1038/srep10788 PMC4649910

[19] Heuss ND, Pierson MJ, Roehrich H, McPherson SW et al (2018) Optic nerve as a source of activated retinal microglia post-injury. Acta Neuropathol Commun 6(1):66. 10.1186/s40478-018-0571-8 30037353 10.1186/s40478-018-0571-8 PMC6055350

[20] Hu X (2020) Microglia/macrophage polarization: fantasy or evidence of functional diversity? J Cereb Blood Flow Metab 40(1_suppl):S134–S136. 10.1177/0271678X20963405 33023387 10.1177/0271678X20963405 PMC7687031

[21] Czeh M, Gressens P, Kaindl AM (2011) The yin and yang of microglia. Dev Neurosci 33(3–4):199–209. 10.1159/000328989 21757877 10.1159/000328989

[22] Tang Y, Le W (2016) Differential roles of M1 and M2 microglia in neurodegenerative diseases. Mol Neurobiol 53(2):1181–1194. 10.1007/s12035-014-9070-5 25598354 10.1007/s12035-014-9070-5

[23] Sas AR, Carbajal KS, Jerome AD, Menon R et al (2020) A new neutrophil subset promotes CNS neuron survival and axon regeneration. Nat Immunol 21(12):1496–1505. 10.1038/s41590-020-00813-0 33106668 10.1038/s41590-020-00813-0 PMC7677206

[24] Zhao X, Sun R, Luo X, Wang F et al (2021) The interaction between microglia and macroglia in glaucoma. Front Neurosci 15:610788. 10.3389/fnins.2021.610788 34121982 10.3389/fnins.2021.610788 PMC8193936

[25] Kang EY, Liu PK, Wen YT, Quinn PMJ et al (2021) Role of oxidative stress in ocular diseases associated with retinal ganglion cells degeneration. Antioxidants (Basel) 10(12):1948. 10.3390/antiox10121948 34943051 10.3390/antiox10121948 PMC8750806

[26] Jurgens HA, Johnson RW (2012) Dysregulated neuronal-microglial cross-talk during aging, stress and inflammation. Exp Neurol 233(1):40–48. 10.1016/j.expneurol.2010.11.014 21110971 10.1016/j.expneurol.2010.11.014 PMC3071456

[27] London A, Itskovich E, Benhar I, Kalchenko V et al (2011) Neuroprotection and progenitor cell renewal in the injured adult murine retina requires healing monocyte-derived macrophages. J Exp Med 208(1):23–39. 10.1084/jem.20101202 21220455 10.1084/jem.20101202 PMC3023128

[28] Tezel G, Yang X, Yang J, Wax MB (2004) Role of tumor necrosis factor receptor-1 in the death of retinal ganglion cells following optic nerve crush injury in mice. Brain Res 996(2):202–212. 10.1016/j.brainres.2003.10.029 14697498 10.1016/j.brainres.2003.10.029

[29] Boyd ZS, Kriatchko A, Yang J, Agarwal N et al (2003) Interleukin-10 receptor signaling through STAT-3 regulates the apoptosis of retinal ganglion cells in response to stress. Invest Ophthalmol Vis Sci 44(12):5206–5211. 10.1167/iovs.03-0534 14638718 10.1167/iovs.03-0534

[30] Bray ER, Yungher BJ, Levay K, Ribeiro M et al (2019) Thrombospondin-1 mediates axon regeneration in retinal ganglion cells. Neuron 103(4):642-657 e7. 10.1016/j.neuron.2019.05.044 31255486 10.1016/j.neuron.2019.05.044 PMC6706310

[31] Tran NM, Shekhar K, Whitney IE, Jacobi A et al (2019) Single-cell profiles of retinal ganglion cells differing in resilience to injury reveal neuroprotective genes. Neuron 104(6):1039-1055 e12. 10.1016/j.neuron.2019.11.006 31784286 10.1016/j.neuron.2019.11.006 PMC6923571

[32] Chu X, Wang C, Wu Z, Fan L et al (2021) JNK/c-Jun-driven NLRP3 inflammasome activation in microglia contributed to retinal ganglion cells degeneration induced by indirect traumatic optic neuropathy. Exp Eye Res 202:108335. 10.1016/j.exer.2020.108335 33141050 10.1016/j.exer.2020.108335

[33] Puyang Z, Feng L, Chen H, Liang P et al (2016) Retinal ganglion cell loss is delayed following optic nerve crush in NLRP3 knockout mice. Sci Rep 6:20998. 10.1038/srep20998 26893104 10.1038/srep20998 PMC4759563

[34] Venanzi AW, Carmy-Bennun T, Marino FS, Ribeiro M et al (2023) Context-dependent effects of the ketogenic diet on retinal ganglion cell (RGC) survival and axonal regeneration after optic nerve injury. JOPT 39:509 10.1089/jop.2023.0001 PMC10616950 37172141

[35] Harun-Or-Rashid M, Pappenhagen N, Palmer PG, Smith MA et al (2018) Structural and functional rescue of chronic metabolically stressed optic nerves through respiration. J Neurosci 38(22):5122–5139. 10.1523/JNEUROSCI.3652-17.2018 29760184 10.1523/JNEUROSCI.3652-17.2018 PMC5977447

[36] Gough SM, Casella A, Ortega KJ, Hackam AS (2021) Neuroprotection by the ketogenic diet: evidence and controversies. Front Nutr 8:782657. 10.3389/fnut.2021.782657 34888340 10.3389/fnut.2021.782657 PMC8650112

[37] Howell GR, Macalinao DG, Sousa GL, Walden M et al (2011) Molecular clustering identifies complement and endothelin induction as early events in a mouse model of glaucoma. J Clin Invest 121(4):1429–1444. 10.1172/JCI44646 21383504 10.1172/JCI44646 PMC3069778

[38] Peterson SL, Li Y, Sun CJ, Wong KA et al (2021) Retinal ganglion cell axon regeneration requires complement and myeloid cell activity within the optic nerve. J Neurosci 41(41):8508–8531. 10.1523/JNEUROSCI.0555-21.2021 34417332 10.1523/JNEUROSCI.0555-21.2021 PMC8513703

[39] Bosco A, Inman DM, Steele MR, Wu G et al (2008) Reduced retina microglial activation and improved optic nerve integrity with minocycline treatment in the DBA/2J mouse model of glaucoma. Invest Ophthalmol Vis Sci 49(4):1437–1446. 10.1167/iovs.07-1337 18385061 10.1167/iovs.07-1337

[40] Grotegut P, Perumal N, Kuehn S, Smit A et al (2020) Minocycline reduces inflammatory response and cell death in a S100B retina degeneration model. J Neuroinflammation 17(1):375. 10.1186/s12974-020-02012-y 33317557 10.1186/s12974-020-02012-y PMC7737388

[41] Rao M, Huang YK, Liu CC, Meadows C et al (2023) Aldose reductase inhibition decelerates optic nerve degeneration by alleviating retinal microglia activation. Sci Rep 13(1):5592. 10.1038/s41598-023-32702-5 37019993 10.1038/s41598-023-32702-5 PMC10076364

[42] Heese K, Hock C, Otten U (1998) Inflammatory signals induce neurotrophin expression in human microglial cells. J Neurochem 70(2):699–707. 10.1046/j.1471-4159.1998.70020699.x 9453564 10.1046/j.1471-4159.1998.70020699.x

[43] Todd L, Palazzo I, Suarez L, Liu X et al (2019) Reactive microglia and IL1beta/IL-1R1-signaling mediate neuroprotection in excitotoxin-damaged mouse retina. J Neuroinflammation 16(1):118. 10.1186/s12974-019-1505-5 31170999 10.1186/s12974-019-1505-5 PMC6555727

[44] Tribble JR, Kastanaki E, Uslular AB, Rutigliani C et al (2022) Valproic acid reduces neuroinflammation to provide retinal ganglion cell neuroprotection in the retina axotomy model. Front Cell Dev Biol 10:903436. 10.3389/fcell.2022.903436 35646919 10.3389/fcell.2022.903436 PMC9135180

[45] Yang P, Chen L, Shi Y, Zhou F et al (2021) Progesterone alters the activation and typing of the microglia in the optic nerve crush model. Exp Eye Res 212:108805. 10.1016/j.exer.2021.108805 34699875 10.1016/j.exer.2021.108805

[46] Yang P, Wei L, Tian H, Yu F et al (2022) Gadolinium chloride protects neurons by regulating the activation of microglia in the model of optic nerve crush. Biochem Biophys Res Commun 618:119–126. 10.1016/j.bbrc.2022.05.088 35717906 10.1016/j.bbrc.2022.05.088

[47] Patel AK, Park KK, Hackam AS (2017) Wnt signaling promotes axonal regeneration following optic nerve injury in the mouse. Neuroscience 343:372–383. 10.1016/j.neuroscience.2016.12.020 28011153 10.1016/j.neuroscience.2016.12.020 PMC5263026

[48] Gao W, Du J, Chi Y, Zhu R et al (2021) Minocycline prevents the inflammatory response after retinal detachment, where microglia phenotypes being regulated through A20. Exp Eye Res 203:108403. 10.1016/j.exer.2020.108403 33326811 10.1016/j.exer.2020.108403

[49] Myhre CL, Thygesen C, Villadsen B, Vollerup J et al (2019) Microglia express insulin-like growth factor-1 in the hippocampus of aged APP(swe)/PS1(DeltaE9) transgenic mice. Front Cell Neurosci 13:308. 10.3389/fncel.2019.00308 31417357 10.3389/fncel.2019.00308 PMC6682662

[50] Ryu KY, Cho GS, Piao HZ, Kim WK (2012) Role of TGF-beta in survival of phagocytizing microglia: autocrine suppression of TNF-alpha production and oxidative stress. Exp Neurobiol 21(4):151–157. 10.5607/en.2012.21.4.151 23319875 10.5607/en.2012.21.4.151 PMC3538179

[51] Lee SC, Liu W, Dickson DW, Brosnan CF et al (1993) Cytokine production by human fetal microglia and astrocytes. Differential induction by lipopolysaccharide and IL-1 beta. J Immunol 150(7):2659–67 8454848

[52] Harada T, Harada C, Kohsaka S, Wada E et al (2002) Microglia-Muller glia cell interactions control neurotrophic factor production during light-induced retinal degeneration. J Neurosci 22(21):9228–9236 12417648 10.1523/JNEUROSCI.22-21-09228.2002 PMC6758038

[53] O’Donnell SL, Frederick TJ, Krady JK, Vannucci SJ et al (2002) IGF-I and microglia/macrophage proliferation in the ischemic mouse brain. Glia 39(1):85–97. 10.1002/glia.10081 12112378 10.1002/glia.10081

[54] Muller A, Hauk TG, Leibinger M, Marienfeld R et al (2009) Exogenous CNTF stimulates axon regeneration of retinal ganglion cells partially via endogenous CNTF. Mol Cell Neurosci 41(2):233–246. 10.1016/j.mcn.2009.03.002 19332123 10.1016/j.mcn.2009.03.002

[55] Klocker N, Kermer P, Weishaupt JH, Labes M et al (2000) Brain-derived neurotrophic factor-mediated neuroprotection of adult rat retinal ganglion cells in vivo does not exclusively depend on phosphatidyl-inositol-3′-kinase/protein kinase B signaling. J Neurosci 20(18):6962–6967. 10.1523/JNEUROSCI.20-18-06962.2000 10995840 10.1523/JNEUROSCI.20-18-06962.2000 PMC6772828

[56] Harder JM, Braine CE, Williams PA, Zhu X et al (2017) Early immune responses are independent of RGC dysfunction in glaucoma with complement component C3 being protective. Proc Natl Acad Sci U S A 114(19):E3839–E3848. 10.1073/pnas.1608769114 28446616 10.1073/pnas.1608769114 PMC5441748

[57] Bosco A, Anderson SR, Breen KT, Romero CO et al (2018) Complement C3-targeted gene therapy restricts onset and progression of neurodegeneration in chronic mouse glaucoma. Mol Ther 26(10):2379–2396. 10.1016/j.ymthe.2018.08.017 30217731 10.1016/j.ymthe.2018.08.017 PMC6171099

[58] Hilla AM, Diekmann H, Fischer D (2017) Microglia are irrelevant for neuronal degeneration and axon regeneration after acute injury. J Neurosci 37(25):6113–6124. 10.1523/JNEUROSCI.0584-17.2017 28539419 10.1523/JNEUROSCI.0584-17.2017 PMC6596505

[59] Siddiqui AM, Sabljic TF, Ball AK (2022) Anatomical location of injected microglia in different activation states and time course of injury determines survival of retinal ganglion cells after optic nerve crush. Int J Neurosci 134:1–23. 10.1080/00207454.2022.2142579 10.1080/00207454.2022.2142579 36371721

[60] Gottlieb A, Toledano-Furman N, Prabhakara KS, Kumar A et al (2022) Time dependent analysis of rat microglial surface markers in traumatic brain injury reveals dynamics of distinct cell subpopulations. Sci Rep 12(1):6289. 10.1038/s41598-022-10419-1 35428862 10.1038/s41598-022-10419-1 PMC9012748

[61] Makinde HM, Just TB, Gadhvi GT, Winter DR et al (2020) Microglia adopt longitudinal transcriptional changes after traumatic brain injury. J Surg Res 246:113–122. 10.1016/j.jss.2019.08.024 31563831 10.1016/j.jss.2019.08.024 PMC6917875

[62] Garces K, Carmy T, Illiano P, Brambilla R et al (2020) Increased neuroprotective microglia and photoreceptor survival in the retina from a peptide inhibitor of myeloid differentiation factor 88 (MyD88). J Mol Neurosci 70(6):968–980. 10.1007/s12031-020-01503-0 32072483 10.1007/s12031-020-01503-0

[63] Orihuela R, McPherson CA, Harry GJ (2016) Microglial M1/M2 polarization and metabolic states. Br J Pharmacol 173(4):649–665. 10.1111/bph.13139 25800044 10.1111/bph.13139 PMC4742299

[64] Cui W, Sun C, Ma Y, Wang S et al (2020) Inhibition of TLR4 induces M2 microglial polarization and provides neuroprotection via the NLRP3 inflammasome in Alzheimer’s disease. Front Neurosci 14:444. 10.3389/fnins.2020.00444 32508567 10.3389/fnins.2020.00444 PMC7251077

[65] Jo M, Kim JH, Song GJ, Seo M et al (2017) Astrocytic orosomucoid-2 modulates microglial activation and neuroinflammation. J Neurosci 37(11):2878–2894. 10.1523/JNEUROSCI.2534-16.2017 28193696 10.1523/JNEUROSCI.2534-16.2017 PMC6596722

[66] Mesquida-Veny F, Del Rio JA, Hervera A (2021) Macrophagic and microglial complexity after neuronal injury. Prog Neurobiol 200:101970. 10.1016/j.pneurobio.2020.101970 33358752 10.1016/j.pneurobio.2020.101970

[67] Yin Y, Cui Q, Li Y, Irwin N et al (2003) Macrophage-derived factors stimulate optic nerve regeneration. J Neurosci 23(6):2284–2293. 10.1523/JNEUROSCI.23-06-02284.2003 12657687 10.1523/JNEUROSCI.23-06-02284.2003 PMC6742044

[68] Guillemin GJ, Brew BJ (2004) Microglia, macrophages, perivascular macrophages, and pericytes: a review of function and identification. J Leukoc Biol 75(3):388–397. 10.1189/jlb.0303114 14612429 10.1189/jlb.0303114

[69] Lipski DA, Dewispelaere R, Foucart V, Caspers LE et al (2017) MHC class II expression and potential antigen-presenting cells in the retina during experimental autoimmune uveitis. J Neuroinflammation 14(1):136. 10.1186/s12974-017-0915-5 28720143 10.1186/s12974-017-0915-5 PMC5516361

[70] Zhao J, Chen M, Xu H (2014) Experimental autoimmune uveoretinitis (EAU)-related tissue damage and angiogenesis is reduced in CCL2(-)/(-)CX(3)CR1gfp/gfp mice. Invest Ophthalmol Vis Sci 55(11):7572–7582. 10.1167/iovs.14-15495 25342612 10.1167/iovs.14-15495

[71] Lazarov-Spiegler O, Solomon AS, Zeev-Brann AB, Hirschberg DL et al (1996) Transplantation of activated macrophages overcomes central nervous system regrowth failure. FASEB J 10(11):1296–1302. 10.1096/fasebj.10.11.8836043 8836043 10.1096/fasebj.10.11.8836043

[72] Cen LP, Ng TK, Liang JJ, Xu C et al (2021) Agonist of growth hormone-releasing hormone enhances retinal ganglion cell protection induced by macrophages after optic nerve injury. Proc Natl Acad Sci U S A 118(28):e1920834118. 10.1073/pnas.1920834118 34244423 10.1073/pnas.1920834118 PMC8285901

[73] Stark DT, Anderson DMG, Kwong JMK, Patterson NH et al (2018) Optic nerve regeneration after crush remodels the injury site: molecular insights from imaging mass spectrometry. Invest Ophthalmol Vis Sci 59(1):212–222. 10.1167/iovs.17-22509 29340649 10.1167/iovs.17-22509 PMC5770179

[74] Greenhalgh AD, Zarruk JG, Healy LM, Baskar Jesudasan SJ et al (2018) Peripherally derived macrophages modulate microglial function to reduce inflammation after CNS injury. PLoS Biol 16(10):e2005264. 10.1371/journal.pbio.2005264 30332405 10.1371/journal.pbio.2005264 PMC6205650

[75] Xie L, Cen LP, Li Y, Gilbert HY et al (2022) Monocyte-derived SDF1 supports optic nerve regeneration and alters retinal ganglion cells’ response to Pten deletion. Proc Natl Acad Sci U S A 119(15):e2113751119. 10.1073/pnas.2113751119 35394873 10.1073/pnas.2113751119 PMC9169637

[76] Liu YF, Liang JJ, Ng TK, Hu Z et al (2021) CXCL5/CXCR2 modulates inflammation-mediated neural repair after optic nerve injury. Exp Neurol 341:113711. 10.1016/j.expneurol.2021.113711 33785307 10.1016/j.expneurol.2021.113711

[77] Cen LP, Luo JM, Zhang CW, Fan YM et al (2007) Chemotactic effect of ciliary neurotrophic factor on macrophages in retinal ganglion cell survival and axonal regeneration. Invest Ophthalmol Vis Sci 48(9):4257–4266. 10.1167/iovs.06-0791 17724215 10.1167/iovs.06-0791

[78] Yin Y, Henzl MT, Lorber B, Nakazawa T et al (2006) Oncomodulin is a macrophage-derived signal for axon regeneration in retinal ganglion cells. Nat Neurosci 9(6):843–852. 10.1038/nn1701 16699509 10.1038/nn1701

[79] Yin Y, Cui Q, Gilbert HY, Yang Y et al (2009) Oncomodulin links inflammation to optic nerve regeneration. Proc Natl Acad Sci U S A 106(46):19587–19592. 10.1073/pnas.0907085106 19875691 10.1073/pnas.0907085106 PMC2780793

[80] Kurimoto T, Yin Y, Habboub G, Gilbert HY et al (2013) Neutrophils express oncomodulin and promote optic nerve regeneration. J Neurosci 33(37):14816–14824. 10.1523/JNEUROSCI.5511-12.2013 24027282 10.1523/JNEUROSCI.5511-12.2013 PMC3771038

[81] Liang JJ, Liu YF, Ng TK, Xu CY et al (2021) Peritoneal macrophages attenuate retinal ganglion cell survival and neurite outgrowth. Neural Regen Res 16(6):1121–1126. 10.4103/1673-5374.300462 33269759 10.4103/1673-5374.300462 PMC8224139

[82] Jin H, Liu Y, Liu X, Khodeiry MM et al (2022) Hematogenous macrophages contribute to fibrotic scar formation after optic nerve crush. Mol Neurobiol 59(12):7393–7403. 10.1007/s12035-022-03052-6 36181661 10.1007/s12035-022-03052-6 PMC10966580

[83] Chen Y, Xia Q, Zeng Y, Zhang Y et al (2022) Regulations of retinal inflammation: focusing on Muller glia. Front Cell Dev Biol 10:898652. 10.3389/fcell.2022.898652 35573676 10.3389/fcell.2022.898652 PMC9091449

[84] Yi H, Patel AK, Sodhi C, Hackam DJ et al (2012) Novel role for the innate immune receptor toll-like receptor 4 (TLR4) in the regulation of the Wnt signaling pathway and photoreceptor apoptosis. PLoS One 7(5):e36560 22615780 10.1371/journal.pone.0036560 PMC3355158

[85] Musada GR, Dvoriantchikova G, Myer C, Ivanov D et al (2020) The effect of extrinsic Wnt/beta-catenin signaling in Muller glia on retinal ganglion cell neurite growth. Dev Neurobiol 80(3–4):98–110. 10.1002/dneu.22741 32267608 10.1002/dneu.22741 PMC7377969

[86] Pernet V, Joly S, Dalkara D, Jordi N et al (2013) Long-distance axonal regeneration induced by CNTF gene transfer is impaired by axonal misguidance in the injured adult optic nerve. Neurobiol Dis 51:202–213. 10.1016/j.nbd.2012.11.011 23194670 10.1016/j.nbd.2012.11.011

[87] Vecino E, Rodriguez FD, Ruzafa N, Pereiro X et al (2016) Glia-neuron interactions in the mammalian retina. Prog Retin Eye Res 51:1–40. 10.1016/j.preteyeres.2015.06.003 26113209 10.1016/j.preteyeres.2015.06.003

[88] de Hoz R, Rojas B, Ramirez AI, Salazar JJ et al (2016) Retinal macroglial responses in health and disease. Biomed Res Int 2016:2954721. 10.1155/2016/2954721 27294114 10.1155/2016/2954721 PMC4887628

[89] Hu X, Zhao GL, Xu MX, Zhou H et al (2021) Interplay between Muller cells and microglia aggravates retinal inflammatory response in experimental glaucoma. J Neuroinflammation 18(1):303. 10.1186/s12974-021-02366-x 34952606 10.1186/s12974-021-02366-x PMC8705189

[90] Van Dyck A, Bollaerts I, Beckers A, Vanhunsel S et al (2021) Muller glia-myeloid cell crosstalk accelerates optic nerve regeneration in the adult zebrafish. Glia 69(6):1444–1463. 10.1002/glia.23972 33502042 10.1002/glia.23972

[91] Rutar M, Natoli R, Provis JM (2012) Small interfering RNA-mediated suppression of Ccl2 in Muller cells attenuates microglial recruitment and photoreceptor death following retinal degeneration. J Neuroinflammation 9:221. 10.1186/1742-2094-9-221 22992301 10.1186/1742-2094-9-221 PMC3546872

[92] Palazzo I, Deistler K, Hoang TV, Blackshaw S et al (2020) NF-kappaB signaling regulates the formation of proliferating Muller glia-derived progenitor cells in the avian retina. Development 147(10):dev183418. 10.1242/dev.183418 32291273 10.1242/dev.183418 PMC7325431

[93] Todd L, Finkbeiner C, Wong CK, Hooper MJ et al (2020) Microglia suppress Ascl1-induced retinal regeneration in mice. Cell Rep 33(11):108507. 10.1016/j.celrep.2020.108507 33326790 10.1016/j.celrep.2020.108507

[94] El-Hodiri HM, Bentley JR, Reske AG, Taylor OB et al (2023) Heparin-binding epidermal growth factor and fibroblast growth factor 2 rescue Muller glia-derived progenitor cell formation in microglia- and macrophage-ablated chick retinas. Development 150(23): dev202070. 10.1242/dev.202070 10.1242/dev.202070 PMC10730090 37971210

[95] Ji M, Sun Q, Zhang G, Huang Z et al (2022) Microglia-derived TNF-alpha mediates Muller cell activation by activating the TNFR1-NF-kappaB pathway. Exp Eye Res 214:108852. 10.1016/j.exer.2021.108852 34801535 10.1016/j.exer.2021.108852

[96] Wang M, Ma W, Zhao L, Fariss RN et al (2011) Adaptive Muller cell responses to microglial activation mediate neuroprotection and coordinate inflammation in the retina. J Neuroinflammation 8:173. 10.1186/1742-2094-8-173 22152278 10.1186/1742-2094-8-173 PMC3251543

[97] Taylor S, Srinivasan B, Wordinger RJ, Roque RS (2003) Glutamate stimulates neurotrophin expression in cultured Muller cells. Brain Res Mol Brain Res 111(1–2):189–197 12654519 10.1016/s0169-328x(03)00030-5

[98] Seki M, Tanaka T, Sakai Y, Fukuchi T et al (2005) Muller cells as a source of brain-derived neurotrophic factor in the retina: noradrenaline upregulates brain-derived neurotrophic factor levels in cultured rat Muller cells. Neurochem Res 30(9):1163–1170 16292510 10.1007/s11064-005-7936-7

[99] Fudalej E, Justyniarska M, Kasarello K, Dziedziak J et al (2021) Neuroprotective factors of the retina and their role in promoting survival of retinal ganglion cells: a review. Ophthalmic Res 64(3):345–355. 10.1159/000514441 33454713 10.1159/000514441

[100] Lin X, Fang D, Zhou H, Su SB (2013) The expression of Toll-like receptors in murine Muller cells, the glial cells in retina. Neurol Sci 34(8):1339–1346. 10.1007/s10072-012-1236-1 23207548 10.1007/s10072-012-1236-1 PMC3747325

[101] Lorenz L, Hirmer S, Schmalen A, Hauck SM et al (2021) Cell surface profiling of retinal Muller glial cells reveals association to immune pathways after LPS stimulation. Cells 10(3):711. 10.3390/cells10030711 33806940 10.3390/cells10030711 PMC8004686

[102] Giblin MJ, Smith TE, Winkler G, Pendergrass HA et al (2021) Nuclear factor of activated T-cells (NFAT) regulation of IL-1beta-induced retinal vascular inflammation. Biochim Biophys Acta Mol Basis Dis 1867(12):166238. 10.1016/j.bbadis.2021.166238 34343639 10.1016/j.bbadis.2021.166238 PMC8565496

[103] Liu X, Ye F, Xiong H, Hu DN et al (2015) IL-1beta induces IL-6 production in retinal Muller cells predominantly through the activation of p38 MAPK/NF-kappaB signaling pathway. Exp Cell Res 331(1):223–231. 10.1016/j.yexcr.2014.08.040 25239226 10.1016/j.yexcr.2014.08.040

[104] Natoli R, Fernando N, Madigan M, Chu-Tan JA et al (2017) Microglia-derived IL-1beta promotes chemokine expression by Muller cells and RPE in focal retinal degeneration. Mol Neurodegener 12(1):31. 10.1186/s13024-017-0175-y 28438165 10.1186/s13024-017-0175-y PMC5404662

[105] Pannicke T, Wagner L, Reichenbach A, Grosche A (2018) Electrophysiological characterization of Muller cells from the ischemic retina of mice deficient in the leukemia inhibitory factor. Neurosci Lett 670:69–74. 10.1016/j.neulet.2018.01.047 29391217 10.1016/j.neulet.2018.01.047

[106] Kirsch M, Trautmann N, Ernst M, Hofmann HD (2010) Involvement of gp130-associated cytokine signaling in Muller cell activation following optic nerve lesion. Glia 58(7):768–779. 10.1002/glia.20961 20091786 10.1002/glia.20961

[107] Leibinger M, Muller A, Andreadaki A, Hauk TG et al (2009) Neuroprotective and axon growth-promoting effects following inflammatory stimulation on mature retinal ganglion cells in mice depend on ciliary neurotrophic factor and leukemia inhibitory factor. J Neurosci 29(45):14334–14341. 10.1523/JNEUROSCI.2770-09.2009 19906980 10.1523/JNEUROSCI.2770-09.2009 PMC6665071

[108] Lebrun-Julien F, Duplan L, Pernet V, Osswald I et al (2009) Excitotoxic death of retinal neurons in vivo occurs via a non-cell-autonomous mechanism. J Neurosci 29(17):5536–5545. 10.1523/JNEUROSCI.0831-09.2009 19403821 10.1523/JNEUROSCI.0831-09.2009 PMC6665839

[109] Hackam AS (2005) The Wnt signaling pathway in retinal degenerations. IUBMB Life 57(6):381–388 16012046 10.1080/15216540500137586

[110] Musada GR, Carmy-Bennun T, Hackam AS (2022) Identification of a novel axon regeneration role for noncanonical Wnt signaling in the adult retina after injury. eNeuro 9(4):ENEURO.0182-22.2022. 10.1523/ENEURO.0182-22.2022 35914928 10.1523/ENEURO.0182-22.2022 PMC9373906

[111] Van Steenwinckel J, Schang AL, Krishnan ML, Degos V et al (2019) Decreased microglial Wnt/beta-catenin signalling drives microglial pro-inflammatory activation in the developing brain. Brain 142(12):3806–3833. 10.1093/brain/awz319 31665242 10.1093/brain/awz319 PMC6906599

[112] Ye X, Smallwood P, Nathans J (2011) Expression of the Norrie disease gene (Ndp) in developing and adult mouse eye, ear, and brain. Gene Expr Patterns 11(1–2):151–155. 10.1016/j.gep.2010.10.007 21055480 10.1016/j.gep.2010.10.007 PMC3061303

[113] Seitz R, Hackl S, Seibuchner T, Tamm ER et al (2010) Norrin mediates neuroprotective effects on retinal ganglion cells via activation of the Wnt/beta-catenin signaling pathway and the induction of neuroprotective growth factors in Muller cells. J Neurosci 30(17):5998–6010. 10.1523/JNEUROSCI.0730-10.2010 20427659 10.1523/JNEUROSCI.0730-10.2010 PMC6632606

[114] Astafurov K, Dong CQ, Panagis L, Kamthan G et al (2014) Complement expression in the retina is not influenced by short-term pressure elevation. Mol Vis 20:140–152 24505213 PMC3913488

[115] Gerhardinger C, Costa MB, Coulombe MC, Toth I et al (2005) Expression of acute-phase response proteins in retinal Muller cells in diabetes. Invest Ophthalmol Vis Sci 46(1):349–357. 10.1167/iovs.04-0860 15623795 10.1167/iovs.04-0860

[116] Cheng L, Bu H, Portillo JA, Li Y et al (2013) Modulation of retinal Muller cells by complement receptor C5aR. Invest Ophthalmol Vis Sci 54(13):8191–8198. 10.1167/iovs.13-12428 24265019 10.1167/iovs.13-12428 PMC3867184

[117] Huxlin KR, Sefton AJ, Furby JH (1992) The origin and development of retinal astrocytes in the mouse. J Neurocytol 21(7):530–544. 10.1007/BF01186955 1500949 10.1007/BF01186955

[118] Paisley CE, Kay JN (2021) Seeing stars: development and function of retinal astrocytes. Dev Biol 478:144–154. 10.1016/j.ydbio.2021.07.007 34260962 10.1016/j.ydbio.2021.07.007 PMC8542354

[119] Yao H, Wang T, Deng J, Liu D et al (2014) The development of blood-retinal barrier during the interaction of astrocytes with vascular wall cells. Neural Regen Res 9(10):1047–1054. 10.4103/1673-5374.133169 25206758 10.4103/1673-5374.133169 PMC4146297

[120] Guttenplan KA, Stafford BK, El-Danaf RN, Adler DI et al (2020) Neurotoxic reactive astrocytes drive neuronal death after retinal injury. Cell Rep 31(12):107776. 10.1016/j.celrep.2020.107776 32579912 10.1016/j.celrep.2020.107776 PMC8091906

[121] Liddelow SA, Guttenplan KA, Clarke LE, Bennett FC et al (2017) Neurotoxic reactive astrocytes are induced by activated microglia. Nature 541(7638):481–487. 10.1038/nature21029 28099414 10.1038/nature21029 PMC5404890

[122] Tang Y, Chen Y, Chen D (2022) The heterogeneity of astrocytes in glaucoma. Front Neuroanat 16:995369. 10.3389/fnana.2022.995369 36466782 10.3389/fnana.2022.995369 PMC9714578

[123] Retamal MA, Froger N, Palacios-Prado N, Ezan P et al (2007) Cx43 hemichannels and gap junction channels in astrocytes are regulated oppositely by proinflammatory cytokines released from activated microglia. J Neurosci 27(50):13781–13792. 10.1523/JNEUROSCI.2042-07.2007 18077690 10.1523/JNEUROSCI.2042-07.2007 PMC6673621

[124] Kawai T, Akira S (2007) TLR signaling. Semin Immunol 19(1):24–32. 10.1016/j.smim.2006.12.004 17275323 10.1016/j.smim.2006.12.004

[125] Singh PK, Kumar A (2015) Retinal photoreceptor expresses toll-like receptors (TLRs) and elicits innate responses following TLR ligand and bacterial challenge. PLoS ONE 10(3):e0119541. 10.1371/journal.pone.0119541 25767877 10.1371/journal.pone.0119541 PMC4358976

[126] Nakano Y, Shimazawa M, Ojino K, Izawa H et al (2017) Toll-like receptor 4 inhibitor protects against retinal ganglion cell damage induced by optic nerve crush in mice. J Pharmacol Sci 133(3):176–183. 10.1016/j.jphs.2017.02.012 28318829 10.1016/j.jphs.2017.02.012

[127] Zhang L, Li X (2020) Toll-like receptor-9 (TLR-9) deficiency alleviates optic nerve injury (ONI) by inhibiting inflammatory response in vivo and in vitro. Exp Cell Res 396(1):112159. 10.1016/j.yexcr.2020.112159 32652081 10.1016/j.yexcr.2020.112159

[128] Luo C, Yang X, Kain AD, Powell DW et al (2010) Glaucomatous tissue stress and the regulation of immune response through glial toll-like receptor signaling. Invest Ophthalmol Vis Sci 51(11):5697–5707. 10.1167/iovs.10-5407 20538986 10.1167/iovs.10-5407 PMC3061506

[129] Lambuk L, Mohd Lazaldin MA, Ahmad S, Iezhitsa I et al (2022) Brain-derived neurotrophic factor-mediated neuroprotection in glaucoma: a review of current state of the art. Front Pharmacol 13:875662. 10.3389/fphar.2022.875662 35668928 10.3389/fphar.2022.875662 PMC9163364

[130] Tan Z, Guo Y, Shrestha M, Sun D et al (2022) Microglia depletion exacerbates retinal ganglion cell loss in a mouse model of glaucoma. Exp Eye Res 225:109273. 10.1016/j.exer.2022.109273 36206859 10.1016/j.exer.2022.109273 PMC10970711

[131] Ma M, Li H, Wu J, Zhang Y et al (2020) Roles of prokineticin 2 in subarachnoid hemorrhage-induced early brain injury via regulation of phenotype polarization in astrocytes. Mol Neurobiol 57(9):3744–3758. 10.1007/s12035-020-01990-7 32572760 10.1007/s12035-020-01990-7

[132] Sun D, Moore S, Jakobs TC (2017) Optic nerve astrocyte reactivity protects function in experimental glaucoma and other nerve injuries. J Exp Med 214(5):1411–1430. 10.1084/jem.20160412 28416649 10.1084/jem.20160412 PMC5413323

[133] Guo MF, Zhang HY, Li YH, Gu QF et al (2020) Fasudil inhibits the activation of microglia and astrocytes of transgenic Alzheimer’s disease mice via the downregulation of TLR4/Myd88/NF-kappaB pathway. J Neuroimmunol 346:577284. 10.1016/j.jneuroim.2020.577284 32652366 10.1016/j.jneuroim.2020.577284

[134] Li X, Li M, Tian L, Chen J et al (2020) Reactive astrogliosis: implications in spinal cord injury progression and therapy. Oxid Med Cell Longev 2020:9494352. 10.1155/2020/9494352 32884625 10.1155/2020/9494352 PMC7455824

[135] Brambilla R, Dvoriantchikova G, Barakat D, Ivanov D et al (2012) Transgenic inhibition of astroglial NF-kappaB protects from optic nerve damage and retinal ganglion cell loss in experimental optic neuritis. J Neuroinflammation 9:213. 10.1186/1742-2094-9-213 22963651 10.1186/1742-2094-9-213 PMC3490907

[136] Chung IY, Benveniste EN (1990) Tumor necrosis factor-alpha production by astrocytes. Induction by lipopolysaccharide, IFN-gamma, and IL-1 beta. J Immunol 144(8):2999–3007 2109008

[137] Hao C, Guilbert LJ, Fedoroff S (1990) Production of colony-stimulating factor-1 (CSF-1) by mouse astroglia in vitro. J Neurosci Res 27(3):314–323. 10.1002/jnr.490270310 2151455 10.1002/jnr.490270310

[138] Mrak RE, Sheng JG, Griffin WS (1995) Glial cytokines in Alzheimer’s disease: review and pathogenic implications. Hum Pathol 26(8):816–823. 10.1016/0046-8177(95)90001-2 7635444 10.1016/0046-8177(95)90001-2 PMC3903413

[139] Fitch MT, Silver J (2008) CNS injury, glial scars, and inflammation: inhibitory extracellular matrices and regeneration failure. Exp Neurol 209(2):294–301. 10.1016/j.expneurol.2007.05.014 17617407 10.1016/j.expneurol.2007.05.014 PMC2268907

[140] Shao WY, Liu X, Gu XL, Ying X et al (2016) Promotion of axon regeneration and inhibition of astrocyte activation by alpha A-crystallin on crushed optic nerve. Int J Ophthalmol 9(7):955–66. 10.18240/ijo.2016.07.04 27500100 10.18240/ijo.2016.07.04 PMC4951673

[141] Charalambous P, Hurst LA, Thanos S (2008) Engrafted chicken neural tube-derived stem cells support the innate propensity for axonal regeneration within the rat optic nerve. Invest Ophthalmol Vis Sci 49(8):3513–3524. 10.1167/iovs.07-1473 18408190 10.1167/iovs.07-1473

[142] Bush TG, Puvanachandra N, Horner CH, Polito A et al (1999) Leukocyte infiltration, neuronal degeneration, and neurite outgrowth after ablation of scar-forming, reactive astrocytes in adult transgenic mice. Neuron 23(2):297–308. 10.1016/s0896-6273(00)80781-3 10399936 10.1016/s0896-6273(00)80781-3

[143] Singh B, Plemel JR (2014) Neutrophil contribution in facilitating optic nerve regeneration. J Neurosci 34(4):1081–1082. 10.1523/JNEUROSCI.4827-13.2014 24453300 10.1523/JNEUROSCI.4827-13.2014 PMC6705301

[144] Xie L, Yin Y, Benowitz L (2021) Chemokine CCL5 promotes robust optic nerve regeneration and mediates many of the effects of CNTF gene therapy. Proc Natl Acad Sci U S A 118(9):e2017282118. 10.1073/pnas.2017282118 33627402 10.1073/pnas.2017282118 PMC7936361

[145] Xing J, Lukomska A, Rheaume BA, Kim J et al (2023) Post-injury born oligodendrocytes incorporate into the glial scar and contribute to the inhibition of axon regeneration. Development 150(8):dev201311. 10.1242/dev.201311 36971369 10.1242/dev.201311 PMC10163352

[146] Domingues HS, Portugal CC, Socodato R, Relvas JB (2016) Oligodendrocyte, astrocyte, and microglia crosstalk in myelin development, damage, and repair. Front Cell Dev Biol 4:71. 10.3389/fcell.2016.00071 27551677 10.3389/fcell.2016.00071 PMC4923166

[147] Wang J, He X, Meng H, Li Y et al (2020) Robust myelination of regenerated axons induced by combined manipulations of GPR17 and microglia. Neuron 108(5):876-886 e4. 10.1016/j.neuron.2020.09.016 33108748 10.1016/j.neuron.2020.09.016 PMC7736523

[148] Cunha MI, Su M, Cantuti-Castelvetri L, Muller SA et al (2020) Pro-inflammatory activation following demyelination is required for myelin clearance and oligodendrogenesis. J Exp Med 217(5):e20191390. 10.1084/jem.20191390 32078678 10.1084/jem.20191390 PMC7201919

[149] Lloyd AF, Davies CL, Holloway RK, Labrak Y et al (2019) Central nervous system regeneration is driven by microglia necroptosis and repopulation. Nat Neurosci 22(7):1046–1052. 10.1038/s41593-019-0418-z 31182869 10.1038/s41593-019-0418-z PMC6597360

[150] Miron VE, Boyd A, Zhao JW, Yuen TJ et al (2013) M2 microglia and macrophages drive oligodendrocyte differentiation during CNS remyelination. Nat Neurosci 16(9):1211–1218. 10.1038/nn.3469 23872599 10.1038/nn.3469 PMC3977045

[151] Olah M, Amor S, Brouwer N, Vinet J et al (2012) Identification of a microglia phenotype supportive of remyelination. Glia 60(2):306–321. 10.1002/glia.21266 22072381 10.1002/glia.21266

